# Calcined Co(II)-Chelated Polyazomethine as Cathode Catalyst of Anion Exchange Membrane Fuel Cells

**DOI:** 10.3390/polym14091784

**Published:** 2022-04-27

**Authors:** Yu-Wei Cheng, Tar-Hwa Hsieh, Yu-Chang Huang, Po-Hao Tseng, Yen-Zen Wang, Ko-Shan Ho, Yue-Jie Huang

**Affiliations:** 1 Department of Chemical Engineering, Ming Chi University of Technology, New Taipei City 243303, Taiwan; louischengblue@gmail.com; 2 Department of Chemical and Materials Engineering, National Kaohsiung University of Science and Technology, 415, Chien-Kuo Road, Kaohsiung 80782, Taiwan; thh@nkust.edu.tw (T.-H.H.); ych@nkust.edu.tw (Y.-C.H.); f108146103@nkust.edu.tw (Y.-J.H.); 3 Graduate Institute of Biomedical Electronics and Bioinformatics, National Taiwan University, 1, Sec. 4, Roosevelt Road, Taipei 10617, Taiwan; d10945009@ntu.edu.tw; 4 Department of Chemical and Materials Engineering, National Yu-Lin University of Science & Technology, 123, Sec. 3, University Road, Dou-Liu City, Yun-Lin 64301, Taiwan

**Keywords:** polyazomethine, cathode catalyst, oxygen reduction reaction, anion exchange membrane fuel cell

## Abstract

Polyazomethine (PAM) prepared from the polycondensation between p-phenylene diamine (PDA) and p-terephthalaldehyde (PTAl) via Schiff reaction can physically crosslink (complex) with Co ions. Co-complexed PAM (Co-PAM) in the form of gel is calcined to become a Co, N-co-doped carbonaceous matrix (Co-N-C), acting as cathode catalyst of an anion exchange membrane fuel cell (AEMFC). The obtained Co-N-C catalyst demonstrates a single-atom structure with active Co centers seen under the high-resolution transmission electron microscopy (HRTEM). The Co-N-C catalysts are also characterized by XRD, SEM, TEM, XPS, BET, and Raman spectroscopy. The Co-N-C catalysts demonstrate oxygen reduction reaction (ORR) activity in the KOH(aq) by expressing an onset potential of 1.19–1.37 V vs. RHE, a half wave potential of 0.70–0.92 V, a Tafel slope of 61–89 mV/dec., and number of exchange electrons of 2.48–3.79. Significant ORR peaks appear in the current–voltage (CV) polarization curves for the Co-N-C catalysts that experience two-stage calcination higher than 900 °C, followed by double acid leaching (CoNC-1000A-900A). The reduction current of CoNC-1000A-900A is comparable to that of commercial Pt-implanted carbon (Pt/C), and the max power density of the single cell using CoNC-1000A-900A as cathode catalyst reaches 275 mW cm^−2^.

## 1. Introduction

Because most metal materials can be easily corroded in an acidic environment, it is necessary to use high-corrosion-resistance and expensive precious metals as fuel cell electrodes. Among the most used electrode materials, the precious metal platinum (Pt) has always been the best catalytic material for fuel cells. However, due to its limited resource reserves and expensive price, we need to replace it with other cheaper, non-precious materials. On the other hand, in an alkaline environment, metallic parts of fuel cells face fewer corrosion problems, although their electrochemical activity is still far less than that of platinum.

Transition metal and nitrogen-doped carbonaceous networks (MNC: metal, nitrogen-doped carbon matrix) are considered a promising non-platinum group material (NPGM) that can possibly replace PGMs with comparable electrochemical activity and better durability. [1,2] Transition metals, which own six coordination sites and form an octahedral structure, acting as the active centers of the cathode catalyst of anion exchange membrane fuel cells (AEMFCs), can accommodate oxygen molecules with two of their coordination sites, leaving the other four empty sites to bond with nitrogen (MN_4_). [3,4,5,6,7] Fe and Co are often chosen as the main transition metals used to build an MNC network (Fe-N-C and Co-N-C, respectively) and behave as the cathode catalyst of the proton exchange membrane fuel cell (PEMFC) or AEMFC [8,9,10,11,12,13,14,15,16,17,18,19].

A carbonaceous matrix can be created by calcining nitrogen-containing (N-containing) aromatic polymers that are able to form robust complexes with either Fe or Co ions. Therefore, an MNC catalyst can be easily prepared just by calcining the metal-chelating, N-doped aromatic polymers. Eventually, a so-called single-atom catalyst (SAC) system is formed in the carbonaceous matrix, with the MNx active centers acting as the adsorption points for O_2_ in the cathode of a fuel cell [20,21,22,23,24,25,26,27,28,29,30,31,32,33,34,35,36,37,38].

Among the N-doped aromatic polymers, polyazomethine (PAM) can be easily prepared via simple reaction in a short polymerization time. At temperature slightly above or equal to room temperature (RT), we can obtain PAM via the Schiff polycondensation reaction between diamine and dialdehyde. The affluent imine groups are able to capture (complex) Co ions and create gel-like composites. In other words, we can actually already construct a similar Co ion-N-complexed structure inside the Co-PAM body after polymerization, which can be easily calcined to become Co-N-C a catalyst with active CoNx centers as a promising cathode catalyst of AEMFC.

In this study, we characterized Co-N-C catalysts with FTIR (Fourier transform infrared), XRD (x-ray diffraction), XPS (x-ray photoelectron spectroscopy), Raman spectroscopy and measured the ORR by polarization curves and the reduction current by LSV (linear sweeping voltage). Eventually, we prepared an MEA (membrane electrode assembly), which was fabricated into a single cell to measure its power density and voltage drop vs. current density.

## 2. Materials and Methods

### 2.1. Materials

PDA (para-phenylene diamine) (Tokyo Kasei Kogyo Co., Ltd., Tokyo, Japan), TPAl (terephthalaldehyde) (Tokyo Kasei Kogyo Co., Ltd., Tokyo, Japan), and anhydrous cobalt(II) chloride (CoCl_2_, J.T. Baker, Radnor, PA, USA) 

### 2.2. Preparation of Co-N-C Catalyst

**Figure 1:**
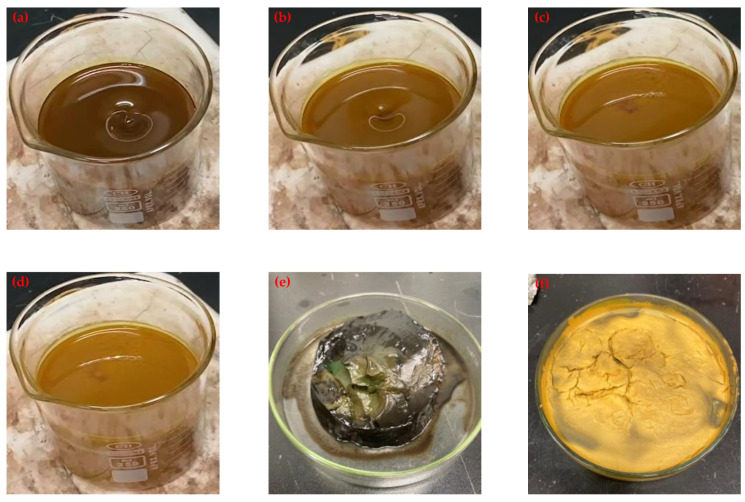
Polymerization mixture of Co-PAM. (**a**) Initial, (**b**) 10 min, (**c**) 20 min, (**d**) 30 min, (**e**) after evaporation of alcohol, (**f**) dry PAM cake prepared without CoCl_2_.

For preparation of the Co-N-C Catalyst, 1.34 g of TPAl in 100 mL alcohol, 1.62 g of PDA in 80 mL alcohol, and 0.99g of CoCl_2_ in 50 mL alcohol were mixed together into one solution. The mixture solution was stirred at room temperature until an orange gel formed, which slowed down the speed of stirring. (Figure 1a–d). The mixture was transferred to a Petri dish, and the alcohol of the gel-like mixture was vaporized at RT, shrinking into a robust jelly gel (Figure 1e). Eventually, the jelly gel was dried at 80^o^C for 8 h before cooling to RT, and Co-PAM was prepared.

The dry, neat PAM prepared in the absence of CoCl_2_ became a dry cake after removing the alcohol, and no gel-like product was obtained (Figure 1f). Dry Co-PAM, the precursor of Co-N-C catalyst, was calcined to 600 °C (700, 800, 900, and 1000 °C) at 10 °C min^−^^1^ and maintained at 600 °C (700, 800, 900, and 1000 °C) for 30 min in an argon atmosphere, then cooled to RT. The impurities and magnetic parts (CoO and Co) of the calcined materials were dissolved by acid leaching with 1 M H_2_SO_4_ (aq.) at 60 °C for 12 h, followed by filtration, and cleansed with deionized water and alcohol before drying in a vacuum oven at 80 °C for 8 hr. The acid-leached products were subjected to a second calcination at 500 °C (600, 700, 800, and 900 °C) in N_2_ and NH_3_ atmospheres at 10 °C min^−^^1^ (named CoNC-600A500) and washed again in 1 M H_2_SO_4_ (aq.) at 60 °C for 30 min, followed by drying in a vacuum oven at 80 °C. The obtained sample was named CoNC-600A-500A (-700A-600A, -800A-700A, -900A-800A, and -1000A-900A).

### 2.3. Characterization

#### 2.3.1. X-ray Photoelectron Spectroscopy (XPS)

Different binding energy spectra of N_1s_ and Co_2p_ belonging to various Co-N-C catalysts were used to characterize the different bonding types of nitrogen and cobalt with a Fison (VG)-Escalab 210 XPS instrument (Fison, Glasgow, UK) using an Al Ka X-ray source at 1486.6 eV. The pressure in the chamber was maintained below 10^−^^6^ Pa. The powered samples were shaped into tablets by a stapler. Binding energies of N_1s_ and Co_2p_ of around 400 and 780 eV, respectively, were recorded.

#### 2.3.2. Raman Spectroscopy

The Raman spectra of all samples were obtained by a Raman spectrometer (TRIAX 320, HOBRIA, Kyoto, Japan).

#### 2.3.3. Wide-angle X-ray Diffraction (WXRD)

A copper (Cu-Kα) Rigaku x-ray source (Rigaku, Tokyo, Japan) with a wavelength of 1.5402 Å was the target for x-ray diffraction. The scanning angle (2θ) ranged from 10° to 90°, with a voltage of 40 kV and a current of 30 mA, operated at 1° min^−1^.

#### 2.3.4. Scanning Electronic Microscopy (SEM)

The sizes and morphologies of Co-N-C catalysts were obtained by SEM (field emission gun scanning electron microscope, AURIGAFE, Zeiss, Oberkochen, Germany).

#### 2.3.5. Transmission Electron Microscopy (TEM)

Photos of the samples were taken using an HR-AEM field emission transmission electron microscope (HITACHI FE-2000, Hitachi, Tokyo, Japan); the samples were first dispersed in acetone and were subsequently placed dropwise on carbonic-coated copper grids before being subjected to electron radiation.

#### 2.3.6. Surface Area and Pore Size Measurement (BET Method)

Nitrogen adsorption–desorption isotherms (type IV) were obtained with an Autosorb IQ gas sorption analyzer (Micromeritics-ASAP2020, Norcross, GA., USA) at 25 °C. The samples were dried in vacuum at a temperature higher than 100 °C overnight. The surface area was calculated according to the BET equation when a linear BET plot with a positive C value was in the relative pressure range. Pore size distribution was determined by the quenched solid density functional theory (QSDFT) method based on a model of slit/cylinder pores. The total pore volumes were determined at P/P_0_ = 0.95.

### 2.4. Electrochemical Characterization

#### 2.4.1. Current–Potential Polarization-Linear Scan Voltammetry (LSV)

Electrocatalyst support was implemented in a three-electrode system. A round working electrode with an area of 1.5 cm^2^ was prepared as follows: Ag/AgCl, carbon graphite, and a Pt strip were used as the reference, relative, and counter electrode, respectively. The electrochemical test was performed in a potentiostat/galvanostat (Autolab-PGSTAT 30 Eco Chemie, KM Utrecht, The Netherlands) in 0.1 M KOH_(aq)_ solution, and C–V curves were obtained from −0.2 to 1.0 V at a scanning rate of 50 mV·s^−^^1^. The catalyst ink was prepared by combining 2.9 mg Co-N-C catalyst powder with a mixture of 375 μL of ethanol and 375 μL of deionized water and stirring until uniform. Subsequently, 7.14 μL of 5% D-2020 Nafion solution (Merck, Darmstadt, Germany) was introduced into the mixture as a binder, the mixture was ultrasonicated for 1 h, and 5μL of the obtained ink was uniformly spray-coated on the carbon paper for C-V testing.

The current–potential polarization curves obtained from the LSVs of the various Co-N-C catalysts were measured using a rotating-disk electrode (RDE: Metrohm, Tampa, FL, USA) operating at 900, 1200, 1600, 2500, and 3600 rpm, respectively, in O_2_-saturated 0.1 M KOH_(aq)_. The reduction current densities of various Co-N-C catalysts, which were recorded at 1600 rpm with 5 mV s^−1^ scanning speed within the measured voltage range (0.0~1.2 V), were chosen for comparison.

#### 2.4.2. MEA Preparation

An X37-50RT sheet (50 μm) purchased from Dioxide Materials, Boca Raton, FL, USA, was used as the hydroxyl ion-exchange membrane. To saturate the membranes with hydroxyl (OH^−^) ions, the X37-50RT (2 × 2 cm) membrane was submerged in 1 M KOH_(aq)_ solution for 24 h. Subsequently, the treated membranes were dipped in distilled water for 15 min and were then stored in 1 M KOH_(aq)_ solution. The catalyst inks were prepared by mixing 18 mg of Co-N-C powders in 400 mg of methanol and 400 mg of deionized water, which were mechanically stirred until uniform, followed by the addition of 90 mg of 5% Sustainion^®^ XB-7 alkaline ionomer ethanol solution (Dioxide Materials, Raton, FL, USA) before stirring again until uniform. Eventually, the catalyst mixture was ultrasonicated for 1 h, followed by dropwise coating on both sides of the treated X37-50RT sheet as the anode (Pt/C) and cathode electrodes (2 × 2 cm), respectively, and hot pressing at 140 °C with a pressure force of 70 kgf cm^−^^2^ for 5 min to obtain the MEA. 

#### 2.4.3. Single-Cell Performance Testing

The MEA was installed in a fuel cell test station to measure the potentials and power densities of the assembled single cell at different current densities using a single-cell testing device (model FCED-P50; Asia Pacific Fuel Cell Technologies, Ltd., Miaoli, Taiwan). The active cell area was 2 × 2 cm. The temperatures of the anode, cell, cathode, and humidifying gas were maintained at about 60 °C. The fuel-flowing rates of the anode input H_2_ and the cathode input O_2_ were set at 30 and 60 mL·min^−^^1^, respectively, based on stoichiometry.

## 3. Results and Discussion

### 3.1. XPS

**Scheme 1:**
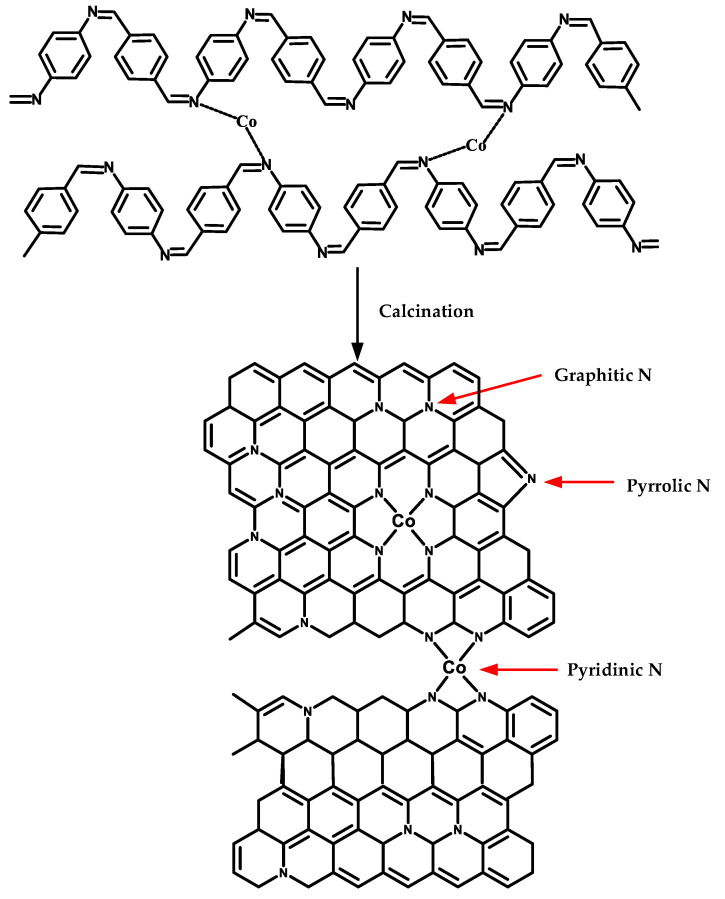
Calcination of Co-PAM into Co-N-C.

**Figure 2:**
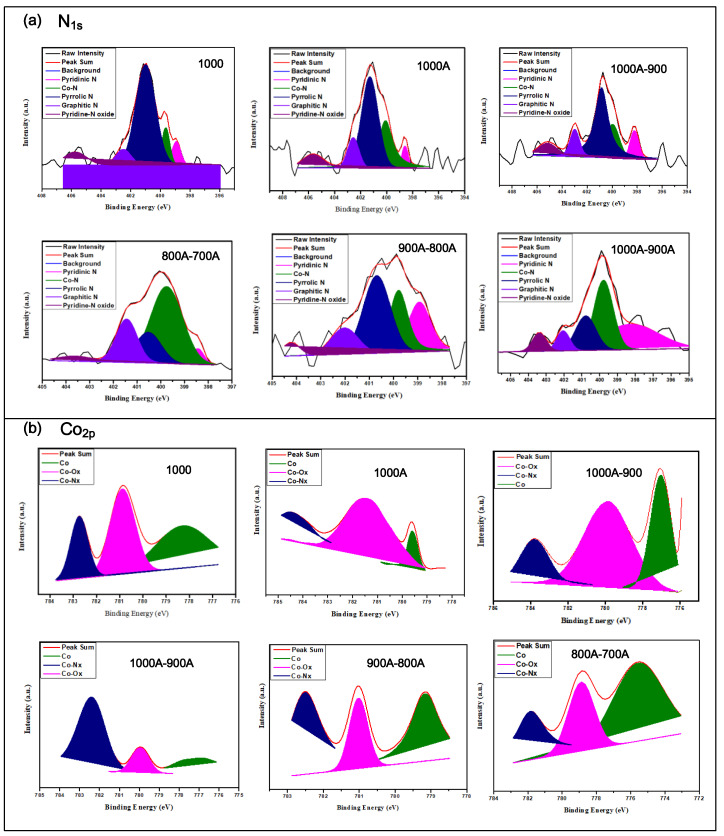
XPS spectra of various Co-N-C catalysts. (**a**) N_1s_, (**b**) Co_2p_.

Nitrogen can bond with carbon, cobalt, and oxygen in various forms, including pyridinic, pyrollic, graphitic, pyridinic oxide-Ns, and Co-N in the matrix of Co-N-C catalysts [39,40,41,42,43], which can be characterized by the XPS of N_1s_ in Figure 2a. The percentages of each type of nitrogen obtained from the deconvolution of XPS are listed in Table 1. We understand that CoNx is the best type of active center for ORR, which can be created in the Co-N-C catalysts after experiencing the 1000A-900A process. Co-Nx demonstrates the highest Co-N composition of 33.82%, as listed in the second column of Table 1. Additionally, it also demonstrates the highest portion of pyridinic N, revealing that Co-N bonding originates from pyridinic Ns that are usually found at the edges of the Co-N-C matrix, as shown in Scheme 1. It seems that the presence of Co-Ns can crosslink the massive carbon matrix together but behaving as the center of catalysis. In investigating the compositions of pyridinic N and Co-N in CoNC-900A-800A, we also found them in higher percentages compared to other types of nitrogen, except CoNC-1000A-900A, in accordance with Table 1, revealing the requirements for the catalyst to experience two-stage calcination and double acid leaching in order to create more active CoNx centers. The contributions of other types of nitrogen seem less important compared to that of pyridinic N.

The chelated Co ions of the Co-PAM might experience reduction, oxidation, and nitridation during high-temperature calcination, possibly resulting producing Co, Co-Ox, and Co-Nx, respectively, which can be quantitatively measured in the XPS of Co_2p_, as illustrated in Figure 2b. The Co metal created on the surface of the catalysts is known to be removed by acid leaching when we compare the spectrum of 1000 with 1000A or 1000A-900 with 1000A-900A in Figure 2b. Some of the Co-Ox, which is considered to be the main source of magnetic attraction of the catalysts, can be neutralized and removed by 1M sulfuric acid (acid treatment), comparing 1000A-900 to 1000A-900A. Although Co-Ox is another possible source of the active catalyzing center of the cathode catalyst, it still needs be removed. Because the Co-O bonding cannot join the carbonaceous network structure, it forms dangling, individual oxides, such as CoO or Co_2_O_3_, in the carbon matrix. Additionally, the catalyst cannot easily disperse in the solvent to prepare the catalyst ink due to the presence of a strong magnetic attraction contributed by Co oxides.

Likewise, the Co metal is able to act as the active center of catalysis of ORR. However, the available surface area for O_2_ is relatively small, and most of the Co atoms inside are not in direct contact with the fuel gas to induce ORR. Co-Nx, which is presented as the SAC in the catalyst network, is the most efficient and active center for ORR in the cathode of the AEMFC.

Briefly, the CoO and Co element found in the XPS spectra are possibly buried deep in the Co-N-C catalysts because the CoO and Co located on the surface can be removed by sulfuric acid and are to induce ORR.

### 3.2. Raman Spectroscopy

**Figure 3:**
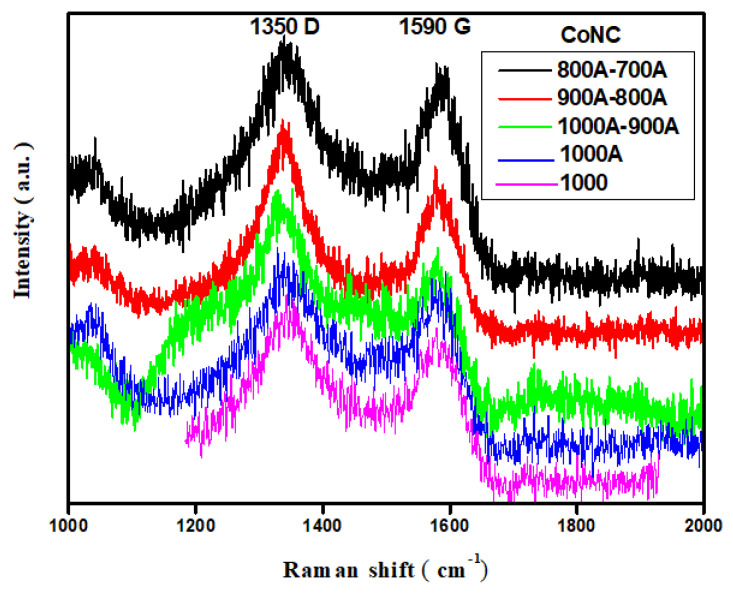
Raman spectra of various Co-N-C catalysts measured from 1000 to 2000 cm^−1^.

The Raman spectrum, which is usually applied to identify the presence of C(sp^3^) and C(sp^2^), can also be used to monitor the surface roughness of the catalyst, with a method similar to that used to calculate the surface area from BET spectra. Carbon materials that experience calcination higher than 800 °C in inert gas are able to develop unsaturated carbons in the form of either a graphene or carbon nanotube (CNT) structure, displaying high concentrations of C(sp^2^) in the Raman spectrum. However, higher concentrations of C(sp^2^) also result in a more plain structure in the conjugated aromatic form, contributing to a smoother surface that is not suitable for a catalyst, which needs high surface area. In other words, we can monitor the surface roughness of the catalyst by comparing the concentration of C(sp^3^) to that of C(sp^2^), which can be achieved by determining the ratio of the intensity of the D band (1350 cm^−1^) and comparing it with that of the G band (1590 cm^−1^), I_D_/I_G_, from the Raman spectrum [16,17]. Figure 3 reveals the D and G bands of various Co-N-C catalysts, and the I_D_/I_G_ values are listed in the first column of Table 2. We understand that the I_D_/I_G_ value is lower for Co-N-C catalysts prepared with one-stage calcination and higher for those prepared with two-stage calcination, indicating many C(sp^2^) remained after the first calcination and more C(sp^2^) converted to C(sp^3^) after the second calcination, resulting in higher I_D_/I_G_ values, as seen in Table 2. Higher concentrations of C(sp^3^) result in more broken surface morphologies of the catalysts, exposing more active Co centers to O_2_ in the cathode.

### 3.3. WAXD Spectroscopy

**Figure 4:**
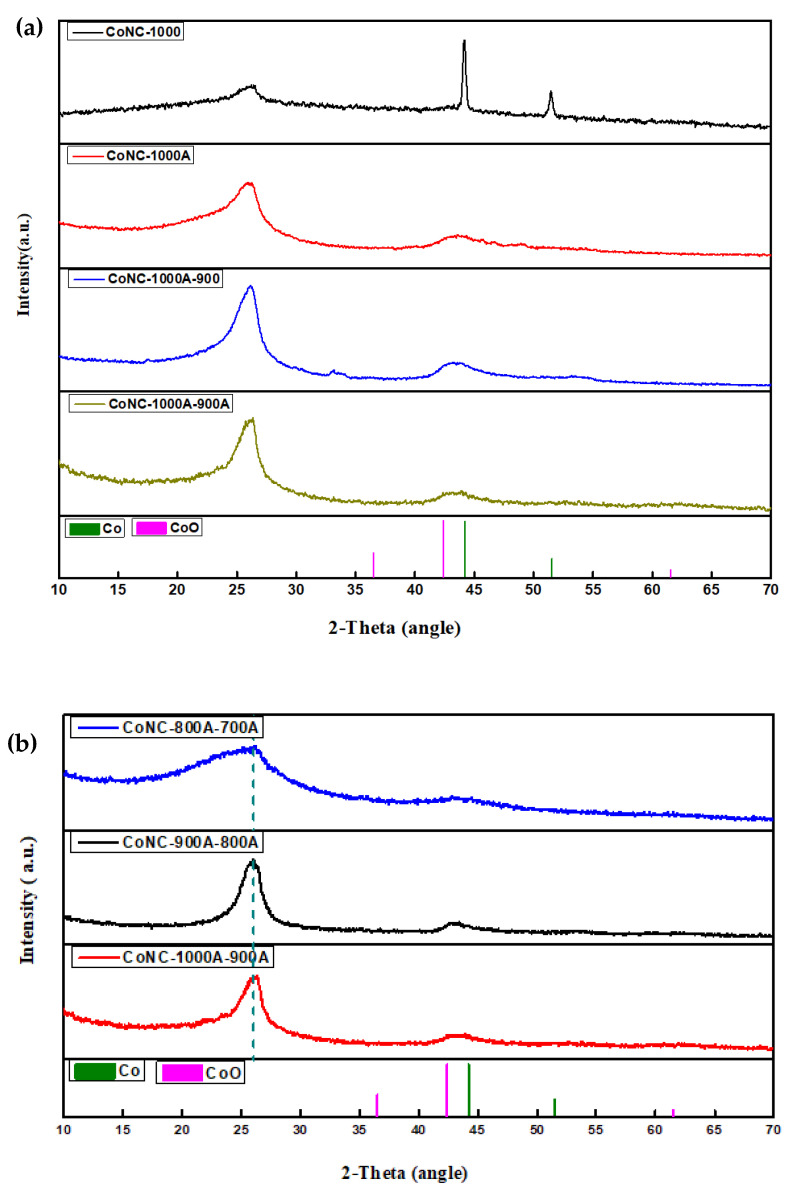
X-ray diffraction patterns of Co-N-C catalysts (**a**) treated at 1000^o^C (**b**) and with two-stage calcination double acid leaching.

The XRD spectra of the Co-N-C catalysts that experienced 1000^o^C calcination are posted in Figure 4a, in which each spectrum demonstrates significant diffraction peaks of C(002) located at around 26° belonging to either graphene or CNT, which provide high conductivity for the transportation of electrons in the electrodes. The significant diffraction peaks of Co(111) and Co(200) at 45° and 52°, respectively, appear in the spectrum of CoNC-1000. Comparing spectra of CoNC-1000 with -1000A, we understand that most of the Co metals formed during calcination can be removed by acid leaching. Small amounts of CoO are present in the Co-N-C catalysts after treatment at 1000 °C.

The XRD spectra of the Co-N-C catalysts prepared with two-stage calcination and double acid-leaching are presented in Figure 4b. No significant C(002) peak is found until the calcination temperature is over 900 °C, and less Co and CoO are found for CoNC-800A-700A.

### 3.4. BET Surface Area and Pore Size Distribution

**Figure 5:**
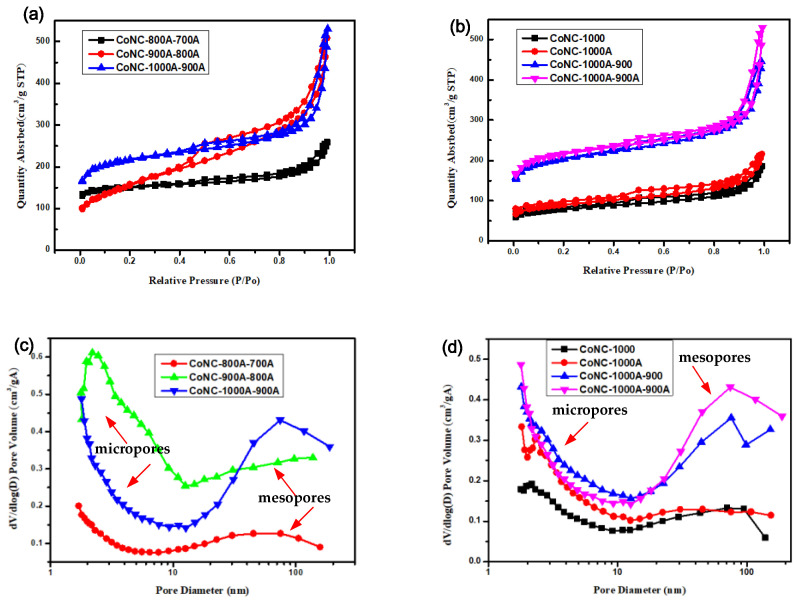
(**a**,**b**) N_2_ adsorption–desorption isotherms and (**c**,**d**) pore size distribution of Co-N-C catalysts treated with different methods of calcination.

The BET surface area significantly increased to more than 450 m^2^g^−1^ (Figure 5a) after the second calcination, which was performed at a temperature 100 °C less than the first calcination in order not to devastate the structure created in the first calcination. A larger amount of ammonia gas compared to N_2_ than that applied in the first calcination was mixed with N_2_ gas in the second calcination to create more and larger pores on the surface of Co-N-C catalysts by efficient bombardment [16,17,19]. This eventually caused a significant increase in surface area, with more mesoporous pores, as presented in the second column of Table 2. The pores newly created in the second calcination demonstrate mesoporous size, as opposed to the microporous structure resulting from the first calcination (Figure 5c,d).

The BET surface area was less than 300 m^2^g^−1^ when only single calcination at 1000 °C was conducted, as presented in the second column of Table 2 and Figure 5d, in which microporous pores dominate (Figure 5d), indicating N_2_ can only construct micropores.

### 3.5. SEM and TEM Micrograph

**Figure 6:**
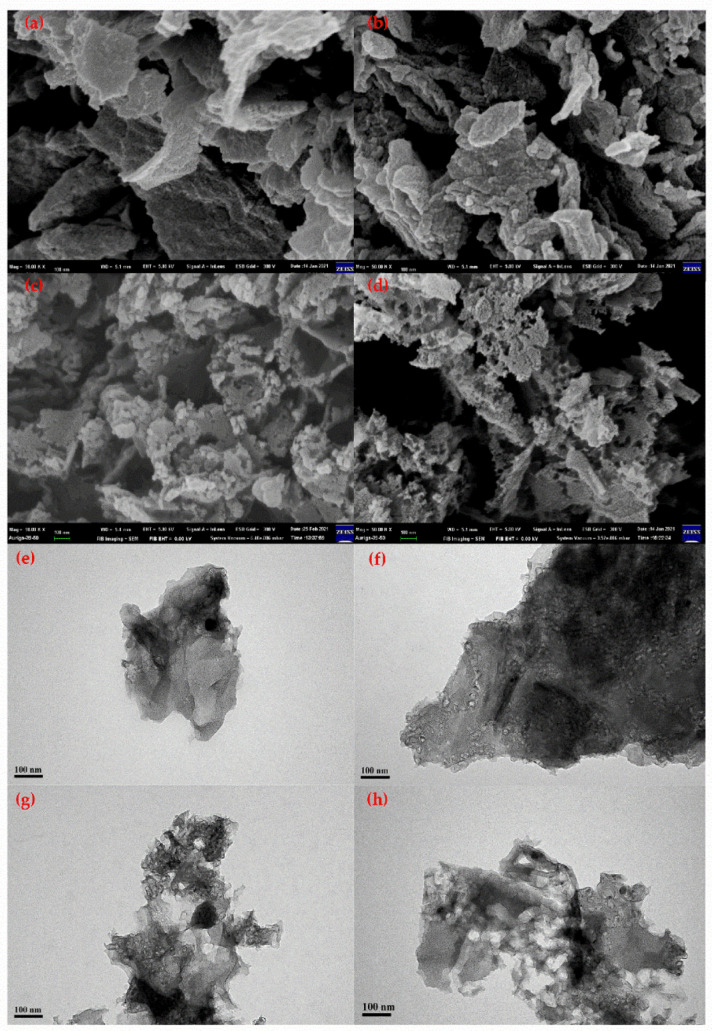
SEM and TEM micrographs of (**a**,**e**) CoNC-1000; (**b**,**f**) CoNC-1000A.;(**c**,**g**) CoNC-1000A-900; and (**d**,**h**) CoNC-1000A-900A.

In order to enhance the capability of pore creation on the surface of the catalyst, Co-PAM was heated to 1000 °C first (CoNC-1000), which resulted in a flake-like morphology (Figure 6a) derived from the formation of a Co, N-doped carbonaceous structure (curved multilayered graphenes) with no significant surface pores seen. After leaching with acid, few micro- and mesoporous pores (based on the data from Figure 5) were perceivable (Figure 6b) for CoNC-1000A, which were created by the vacancies left by the Co elements or CoO washed away by acid. Additional microporous pores are created if the catalyst is subjected to second heating at 900 °C with NH_3_ gas included in the influx mixture gas. In this case, micropores dominate on the surface (Figure 6c), in accordance with Figure 5. Additional mesopores formed when following washing with acid, as illustrated in Figure 6d (CoNC-1000A-900A), resulting in a BET surface area close to 680 m^2^ g^−1^, as presented in Table 2, as more CoNx active centers can are exposed to the incoming O_2_ in the cathode. Briefly, both two-stage heating in different types of gas (N_2_ and NH_3_) and double acid leaching are required to achieve a high concentration of micro- or mesoporous pores on the Co-N-C catalysts.

Similar morphologies were found for TEM micrographs; no significant pores were seen on the surface of CoNC-1000 (Figure 6e) until it was acid-leached (CoNC-1000A in Figure 6f). More tiny pores are perceivable after the second calcination at 900 °C (CoNC-1000A-900 in Figure 6g), and many more pores formed after second acid leaching for CoNC-1000A-900A, as seen in Figure 6h.

### 3.6. Electrochemical Measurement

#### 3.6.1. CV and LSV Curves

**Figure 7:**
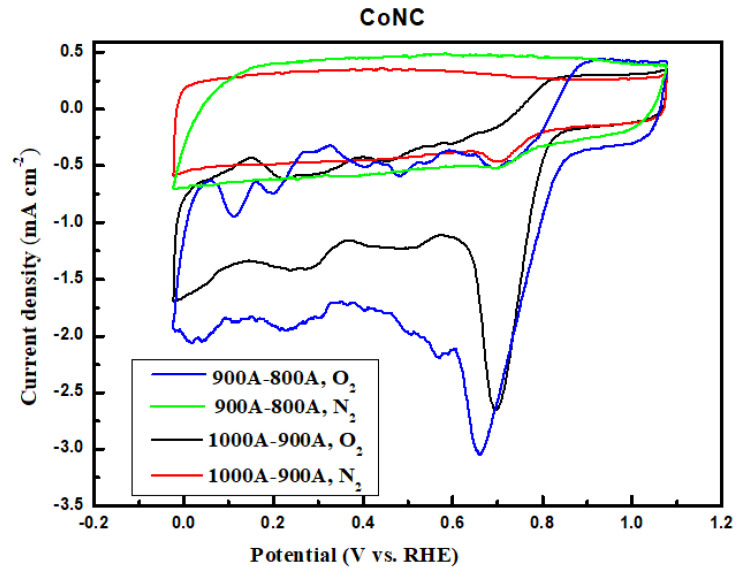
Polarization curves of CoNC 1000A-900A and 900A-800A in N_2_ and O_2_ atmospheres.

The catalyzing capability of the prepared cathode catalyst was characterized first by identifying its reduction peak in the polarization curves (C-V curves). Both CoNC-1000A-900A and -900A-800A were taken at a scanning rate of 50 mV·s^−^^1^, and neither demonstrated a significant reduction peak in an N_2_ atmosphere (Figure 7). However, strong reduction peaks appeared when N_2_ was replaced with O_2_ for both catalysts, revealing both as promising cathode catalysts for ORR in base medium (KOH_(aq)_) and as suitable catalysts of AEMFC.

**Figure 8:**
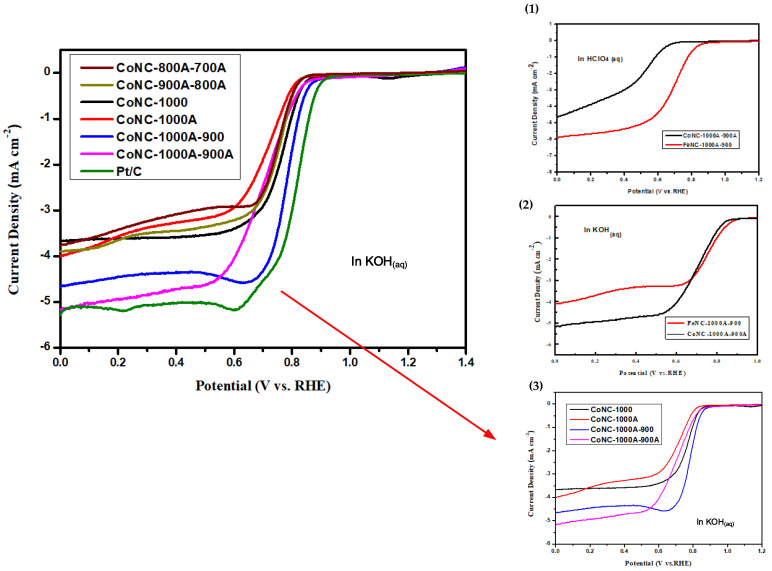
LSV curves of various Co-N-C catalysts. LSV curves of Fe-N-C and Co-N-C in (**1**) HClO_4_, (**2**) KOH, and (**3**) Co-N-C catalysts at 1000 °C.

To further examine the ORR capability of the Co-N-C catalysts, the electrode was replaced by a rotating disk electrode (RDE), and the LSV curves of Pt/C and Co-N-C catalysts were recorded in O_2_-saturated 0.1 M KOH(aq) (or 0.1 M HClO4 (aq) for comparison) solution at 5 mV s^−1^ and 1600 rpm. The reduction current density from 0 to 1 V was recorded for all samples. The limiting reduction current density (LRCD) at 0 V was used as the standard to identify suitable Co-N-C catalysts to fabricate MEA for single-cell testing. In acid medium, more hydrogen peroxide (H_2_O_2_) was produced [44] due to insufficient ORR (two-electron route reduction), which can carry out redox reaction (Fenton process) with Co(II), resulting in the formation of a hydroxyl radical (OH^.^), which can effectively destroy the Co-N-C structure during operation of the PEMFC. The same catalyst (CoNC-1000A-900A) had a lower LRCD (4.6 mA cm^−2^) in the acid medium (Figure 8, inset (1)) in comparison with that obtained in base medium (5.2 mA cm^−2^ (Figure 8, inset (2)), which can be attributed to the damage to Co-N-C structure caused by the generating OH^.^ through the Fenton process in acid medium. On the contrary, less H_2_O_2_ was generated (four-electron route of ORR) in the acid medium when Co-N-C was replaced with Fe-N-C, with LRCD approaching 6 mA cm^−2^ [17] (Figure 8, inset (1)). It seems that the Fe-N-C structure can tolerate the corrosion of hydroxyl radicals.

When we compare the LRCD of all Co-N-C catalysts that experienced 1000 °C in (Figure 8 inset-(3)), we understand that second stage calcination and second acid leaching are necessary for the Co-N-C catalysts to obtain high LRCD. Only Co-PAM prepared with two-stage heating (calcination temperature higher than 900 °C) and washed twice with acid can obtain an LRCD that is comparable to that of commercial Pt/C (Figure 8). The onset and half-wave potentials of various Co-N-C catalysts are also listed in Table 3, with similar values for all catalysts (61–77 mV/dec), except CoNC-1000.

**Figure 9:**
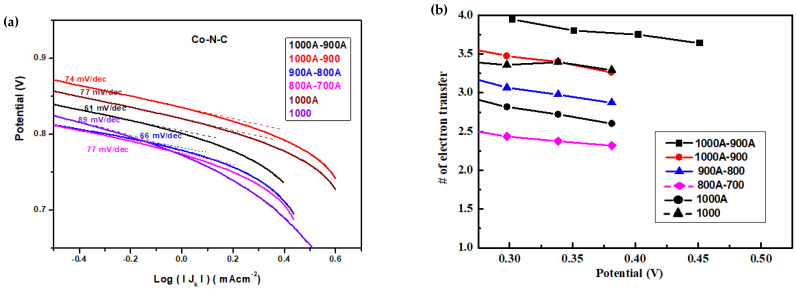
(**a**) Tafel plot derived from LSV curves and (**b**) number of electrons transferred during ORR for various Co-N-C catalysts.

The kinetic current density (J_k_) of LSV curves of various Co-N-C catalysts measured at 1600 rpm in KOH_(aq)_ were used to create the Tafel plot in Figure 9a. The voltage vs. Log (| J_k_ |) diagram is similar to voltage vs. current. Consequently, the Tafel slope obtained from Figure 9a can be considered the resistance of electron transportation in the cathode. The obtained Tafel slopes (Table 3) for all Co-N-C catalysts are smaller than those reported in the literature [45,46,47,48], indicating the e-transferred speed inside the Co-N-C catalysts are faster than that of common Fe-N-C or Co-N-C catalysts. It seems that the resistance of e-transferred speed in the cathode is high if the cathode catalyst does not experience acid leaching, even though it was already calcined at temperatures as high as 1000 °C, comparing the Tafel slope of CoNC-1000 (89 mV/dec) to that of CoNC-1000A (77 mV/dec) in Figure 9a and Table 3. The slope value continued to decreased with second calcination and second acid-leaching, as presented in Table 3, indicating that lower resistance (smaller slope) and higher e-transferring speed can be achieved if the catalysts experience high-temperature (>900 °C) calcination and double acid leaching.

LSV curves measured at different rotational speeds were used to plot the Koutecký–Levich (K-L) lines, which can be used to calculate the number of electrons (n) transferred during ORR according to Koutecký–Levich equation (Figure 9b). The obtained averaged n-values are listed in the third column of Table 3. Both CoNC-1000A-900A and -1000A-900 demonstrated higher numbers of electrons transferred at all potentials and n-values close to 4, indicating more 4e routes were followed during ORR.

#### 3.6.2. MEA and Single Cells

The anode catalysts of all MEAs were made of commercial Pt/C, and the cathode catalysts were Co-N-C catalysts, except for the comparison MEA, in which both anode and cathode catalysts were Pt/C.

**Figure 10:**
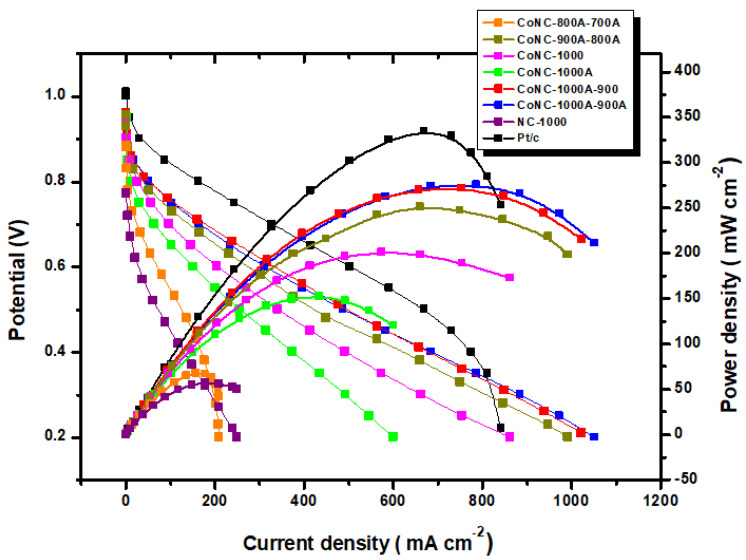
Power density and potential vs. current density of various single cells.

The discussion about the obtained LRCDs of various types of Co-N-C catalysts in Figure 8 reveals that catalysts experienced two-stage calcination, and the process of double acid leaching resulted in a significant reduction in ORR. The power density and potential vs. current density of all single cell prepared with different types of cathode catalysts are illustrated in Figure 10.

For a single cell made of a neat PAM catalyst that prepared at 1000 °C in the absence of Co (NC-1000), the max power density (P_max_) is very low. However, the P_max_ can be increased to close to 200 mW cm^−2^ (CoNC-1000) if the Co is chelated with the precursor (Co-PAM) before calcination.

Most of the obtained Co-N-C catalysts have a Pmax higher than 200 mW cm^−2^, which is not common single cells of AEMFCs using Co-N-C composites as cathode catalysts. The literature [37,49,50,51,52] reports that the P_max_ of single cells based on Co-N-C catalysts is almost always below 200 mW cm^−2^ if the anode is fabricated with 20% Pt/C rather than 40% Pt/C or 20% RuPt/C.

The calcination temperature needs to be higher than 800 °C in order to obtain a single cell with a P_max_ over 200 mW cm^−2^, especially for a single cell using CoNC-1000A-900A as the cathode catalyst, the P_max_ of which can reach 275 mW cm^−2^. The high-power density of single cells using Co-N-C cathode catalysts experiencing high temperature calcination (>900 °C) can be attributed to the presence of CoNx (SAC), CoO, and Co elements, in accordance with the Co_2p_ of XPS in Figure 2b. However, only the exposed surface of both CoO and Co can perform catalysis, not the enclosed ones, and they can be easily removed by acid leaching. Consequently, high concentrations of CoO and Co found in XPS do not mean the cathode catalysts can promote more ORRs because most of them are inside the Co-N-C catalysts and not able to directly interact with O_2_.

Although the P_max_ of all Co-N-C catalysts are lower compared to that of Pt/C catalysts (350 mW cm^−2^), their potentials can extend to more than 1000 mW cm^−2^ (current intensity), and the max current density (I_max_) can be over 900 mW cm^−2^. However, the potential of cells made of Pt/C decays very quickly, and the I_max_ of Pt/C is just 700 mW cm^−2^, indicating the durability of the Pt/C catalyst might not be as favorable as that of Co-N-C catalysts, which will be discussed in the next section.

#### 3.6.3. Durability Testing by Limited Reduction Current Density

Durability testing was conducted in the presence of 0.1 M KOH_(aq)_ by measuring the limited reduction current density (LRCD) after performing multiple redox cycles.

**Figure 11:**
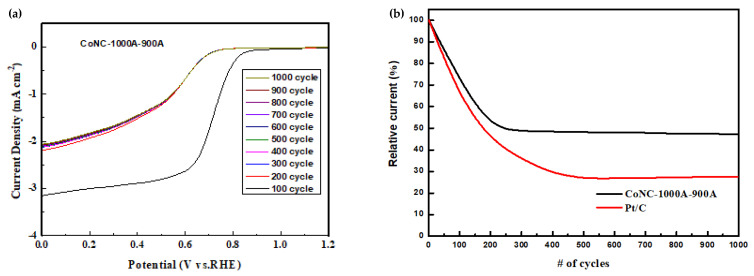
Durability testing of CoNC-1000A-900A and commercial Pt/C catalyst.(**a**) LSV curves obtained with different numbers of cycles. (**b**) Relative current vs. number of cycles derived from (**a**).

The LSV curves of CoNC-1000A-900A after performing different numbers of redox cycles are plotted in Figure 11a, and the relative current is obtained from the ratio of the LRCD, comparing with the first cycle vs. numbers of cycles in Figure 11b to illustrate the durability of the catalyst under continuous, cyclic redox reaction.

We clearly see only 50% decay for CoNC-1000A-900A, and it becomes stable after 300 cycles (Figure 11b). However, the relative ratio of Pt/C decayed to more than 70% after 500 cycles, which confirms that the potential of the single cell made of Pt/C decayed very fast with current density (Figure 10).

## 4. Conclusions

Co ions firmly chelate with the imine groups of PAM to create a gel after polymerization. After calcination, a SAC based on CoNx bonding in the carbonaceous matrix is formed and can effectively improve the ORR in the cathode. The obtained Co-N-C catalysts can enhance the capability of ORR if the calcination was conducted in two-stage heating in different atmospheres plus double acid leaching. High-temperature calcination (>900 °C) can promote the formation of SACs based on CoNx, and acid leaching is able to remove both the CoO compounds and the exposed Co metals on the surface of SACs, leaving many of meso- or micropores large enough for O_2_ molecules enter.

The prepared Co-N-C catalysts demonstrate effective ORR activity in the KOH_(aq)_ by demonstrating an onset potential of 1.19–1.37 V vs. RHE, a half-wave potential of 0.70–0.92 V, a Tafel slope of 61–89 mV/dec., and 2.48–3.79 exchange electrons.

Eventually, we obtained a Co-N-C catalyst that can produce a limited reduction current comparable to that of commercial Pt/C. A single cell based on the MEA using CoNC-1000A-900A as the cathode catalyst demonstrated a max power density of 275 mW cm^−2^ in KOH_(aq)_ medium, which is relatively high compared with that of common AEMFC.

## Figures and Tables

**Table 1:** Atomic ratios of the different N species in Co-N-C-catalysts, as determined by XPS analysis.

CoNC-Catalyst	N_1s_ (at%)				
	Pyridinic N	Co-N	Pyrrolic N	Graphitic N	Pyridine-N oxide
1000	6.6%	9.7%	66.2%	7.8%	9.7%
1000A	4.2%	30.2%	46.9%	10.4%	8.3%
1000A-900	9.76%	18.05%	52.7%	10.71%	8.78%
1000A-900A	35.97%	33.82%	17.48%	6.51%	6.22%
900A-800A	22.15	24.62	36.80	12.46	3.97
800A-700A	1.31	16.60	75.09	5.94	1.06

**Table 2:** Comparison of various properties of Co-N-C catalysts.

Co-N-C-Catalyst	I_D_/I_G_^a^	BET Surface Area (m^2^ g^−1^)	Pore Diameter ^b^
1000	1.07	253.25	4.53
1000A	1.08	290.73	4.58
1000A-900	-	646.85	4.28
1000A-900A	1.12	680.94	4.82
900A-800A	1.14	461.47	5.74
800A-700A	1.08	547.39	3.46

^a^: obtained from Raman spectra. ^b^: obtained from BET adsorption curves.

**Table 3:** Comparison of various electrochemical properties of all Co-N-C catalysts.

CoNC-Catalyst	Onset Potential (V) ^a^	Half-Wave Voltage (V) ^a^	Tafel Slope (mVdec^−1^) ^b^	# of Exchange Electrons ^c^	P_max_ (mWcm^−2^) ^d^
1000	1.37	0.76	89	3.38	200
1000A	1.28	0.92	77	2.75	150
1000A-900	1.26	0.70	74	3.49	270
1000A-900A	1.28	0.78	61	3.79	275
900A-800A	1.25	0.74	66	3.13	250
800A-700A	1.19	0.74	77	2.48	66

^a^: obtained from LSV curves in Figure 9. ^b^: obtained from curves in Figure 10a. ^c^: obtained from curves in Figure 10b. ^d^: obtained from Figure 11.

## Data Availability

The data presented in this study are available upon request from the corresponding author.

## References

[1] Ganesan S., Leonard N., Calabrese Barton S. (2013). Influence of Transition Metal on Oxygen Reduction Activity of Metal-Nitrogen-Carbon Electrocatalyst. ECS Trans..

[2] Tylus U., Jia Q., Strickland K., Ramaswamy N., Serov A., Atanassov P., Mukerjee S. (2014). Elucidating Oxygen Reduction Active Sites in Pyrolyzed Metal–Nitrogen Coordinated Non-Precious-Metal Electrocatalyst Systems. J. Phys. Chem. C.

[3] Wang Y.-Z., Huang W.-Y., Hsieh T.-H., Jheng L.-C., Ho K.-S., Huang S.-W., Chao L. (2019). FeNxC Based Catalysts Prepared by the Calcination of Iron-Ethylenediamine@Polyaniline as the Cathode-Catalyst of Proton Exchange Membrane Fuel Cell. Polymers.

[4] Miao Z., Xia Y., Liang J., Xie L., Chen S., Li S., Wang H.-L., Hu S., Han J., Li Q. (2021). Constructing Co–N–C Catalyst via a Double Crosslinking Hydrogel Strategy for Enhanced Oxygen Reduction Catalysis in Fuel Cells. Small.

[5] Chen P.-W., Li K., Yu Y.-X., Zhang W.-D. (2017). Cobalt-doped graphitic carbon nitride photocatalysts with high activity for hydrogen evolution. Appl. Surf. Sci..

[6] Venegas R., Recio F.J., Zuñiga C., Viera M., Oyarzún M.-P., Silva N., Neira K., Marco J.F., Zagal J.H., Tasca F. (2017). Comparison of the catalytic activity for O_2_ reduction of Fe and Co MN4 adsorbed on graphite electrodes and on carbon nanotubes. Phys. Chem. Chem. Phys..

[7] Zhang G., Huang C., Wang X. (2015). Dispersing Molecular Cobalt in Graphitic Carbon Nitride Frameworks for Photocatalytic Water Oxidation. Small.

[8] Ge X., Sumboja A., Wuu D., An T., Li B., Goh F.W.T., Hor T.S.A., Zong Y., Liu Z. (2015). Oxygen Reduction in Alkaline Media: From Mechanisms to Recent Advances of Catalysts. ACS Catal..

[9] Li J., Jaouen F. (2018). Structure and activity of metal-centered coordination sites in pyrolyzed metal–nitrogen–carbon catalysts for the electrochemical reduction of O_2_. Curr. Opin. Electrochem..

[10] Li T., Chen D., Gu L., Chen S., Li C., Liao J., Zhou Y., Xu Y., Sun C., Yang Z. (2019). Single-source precursor synthesis of nitrogen-doped porous carbon for high-performance electrocatalytic ORR application. Ceram. Int..

[11] Asset T., Atanassov P. (2020). Iron-Nitrogen-Carbon Catalysts for Proton Exchange Membrane Fuel Cells. Joule.

[12] Song M., Song Y., Sha W., Xu B., Guo J., Wu Y. (2020). Recent Advances in Non-Precious Transition Metal/Nitrogen-doped Carbon for Oxygen Reduction Electrocatalysts in PEMFCs. Catalysts.

[13] Marshall-Roth T., Libretto N.J., Wrobel A.T., Anderton K.J., Pegis M.L., Ricke N.D., Voorhis T.V., Miller J.T., Surendranath Y. (2020). A pyridinic Fe-N4 macrocycle models the active sites in Fe/N-doped carbon electrocatalysts. Nat. Commun..

[14] Lilloja J., Kibena-Põldsepp E., Sarapuu A., Kodali M., Chen Y., Asset T., Käärik M., Merisalu M., Paiste P., Aruväli J. (2020). Cathode Catalysts Based on Cobalt- and Nitrogen-Doped Nanocarbon Composites for Anion Exchange Membrane Fuel Cells. ACS Appl. Energy Mater..

[15] Chen M.X., Tong L., Liang H.W. (2021). Understanding the Catalytic Sites of Metal-Nitrogen-Carbon Oxygen Reduction Electrocatalysts. Chem. A Eur. J..

[16] Huang W.Y., Jheng L.C., Hsieh T.H., Ho K.S., Wang Y.Z., Gao Y.J., Tseng P.H. (2020). Calcined Co(II)-Triethylenetetramine, Co(II)-Polyaniline-Thiourea as the Cathode Catalyst of Proton Exchanged Membrane Fuel Cell. Polymers.

[17] Cheng Y.W., Huang W.Y., Ho K.S., Hsieh T.H., Jheng L.C., Kuo Y.M. (2021). Fe, N-Doped Metal Organic Framework Prepared by the Calcination of Iron Chelated Polyimines as the Cathode-Catalyst of Proton Exchange Membrane Fuel Cells. Polymers.

[18] Feng J., Cai R., Magliocca E., Luo H., Higgins L., Romario G.L.F., Liang X., Pedersen A., Xu Z., Guo Z. (2021). Iron, Nitrogen Co-Doped Carbon Spheres as Low Cost, Scalable Electrocatalysts for the Oxygen Reduction Reaction. Adv. Funct. Mater..

[19] Hsieh T.H., Chen S.N., Wang Y.Z., Ho K.S., Chuang J.K., Ho L.C. (2022). Cobalt-Doped Carbon Nitride Frameworks Obtained from Calcined Aromatic Polyimines as Cathode Catalyst of Anion Exchange Membrane Fuel Cells. Membranes.

[20] Wang A., Li J., Zhang T. (2018). Heterogeneous single-atom catalysis. Nat. Rev. Chem..

[21] Zhang L., Ren Y., Liu W., Wang A., Zhang T. (2018). Single-atom catalyst: A rising star for green synthesis of fine chemicals. Natl. Sci. Rev..

[22] Cheng N., Zhang L., Doyle-Davis K., Sun X. (2019). Single-Atom Catalysts: From Design to Application. Electrochem. Energy Rev..

[23] Bai L., Hsu C.-S., Alexander D.T.L., Chen H.M., Hu X. (2019). A Cobalt–Iron Double-Atom Catalyst for the Oxygen Evolution Reaction. J. Am. Chem. Soc..

[24] Cui L., Cui L., Li Z., Zhang J., Wang H., Lu S., Xiang Y. (2019). A copper single-atom catalyst towards efficient and durable oxygen reduction for fuel cells. J. Mater. Chem. A.

[25] Peng X., Omasta T.J., Magliocca E., Wang L.G., Varcoe J.R., Mustain W.E. (2019). Nitrogen-doped Carbon–CoOx Nanohybrids: A Precious Metal Free Cathode that Exceeds 1.0 W cm^−2^ Peak Power and 100 h Life in Anion-Exchange Membrane Fuel Cells. Angew. Chem..

[26] Yang H., Li Z., Kou S., Lu G., Liu Z. (2020). A complex-sequestered strategy to fabricate Fe single-atom catalyst for efficient oxygen reduction in a broad pH-range. Appl. Catal. B Environ..

[27] Kaiser S.K., Chen Z., Faust Akl D., Mitchell S., Perez-Ramirez J. (2020). Single-Atom Catalysts across the Periodic Table. Chem. Rev..

[28] Han J., Bian J., Sun C. (2020). Recent Advances in Single-Atom Electrocatalysts for Oxygen Reduction Reaction. Research.

[29] Zhong L., Li S. (2020). Unconventional Oxygen Reduction Reaction Mechanism and Scaling Relation on Single-Atom Catalysts. ACS Catal..

[30] Zhao C.X., Li B.Q., Liu J.N., Zhang Q. (2021). Intrinsic Electrocatalytic Activity Regulation of M–N–C Single-Atom Catalysts for the Oxygen Reduction Reaction. Angew. Chem. Int. Ed..

[31] Freitas W.d.S., D’Epifanio A., Ficca V.C.A., Placidi E., Arciprete F., Mecheri B. (2021). Tailoring active sites of iron-nitrogen-carbon catalysts for oxygen reduction in alkaline environment: Effect of nitrogen-based organic precursor and pyrolysis atmosphere. Electrochim. Acta.

[32] Guo J., Li B., Zhang Q., Liu Q., Wang Z., Zhao Y., Shui J., Xiang Z. (2021). Highly Accessible Atomically Dispersed Fe-N x Sites Electrocatalyst for Proton-Exchange Membrane Fuel Cell. Adv. Sci..

[33] Deng Y., Luo J., Chi B., Tang H., Li J., Qiao X., Shen Y., Yang Y., Jia C., Rao P. (2021). Advanced Atomically Dispersed Metal–Nitrogen–Carbon Catalysts Toward Cathodic Oxygen Reduction in PEM Fuel Cells. Adv. Energy Mater..

[34] Zhang A., Zhou M., Liu S., Chai M., Jiang S. (2021). Synthesis of Single-Atom Catalysts Through Top-Down Atomization Approaches. Front. Catal..

[35] Liang X., Li Z., Xiao H., Zhang T., Xu P., Zhang H., Gao Q., Zheng L. (2021). Two Types of Single-Atom FeN_4_ and FeN_5_ Electrocatalytic Active Centers on N-Doped Carbon Driving High Performance of the SA-Fe-NC Oxygen Reduction Reaction Catalyst. Chem. Mater..

[36] Kisand K., Sarapuu A., Danilian D., Kikas A., Kisand V., Rähn M., Treshchalov A., Käärik M., Merisalu M., Paiste P. (2021). Transition metal-containing nitrogen-doped nanocarbon catalysts derived from 5-methylresorcinol for anion exchange membrane fuel cell application. J. Colloid Interface Sci..

[37] Zhang J., Pei Y., Zhu W., Liu Y., Yin Y., Qin Y., Guiver M.D. (2021). Ionomer dispersion solvent influence on the microstructure of Co–N–C catalyst layers for anion exchange membrane fuel cell. J. Power Sources.

[38] Adabi H., Santori P.G., Shakouri A., Peng X., Yassin K., Rasin I.G., Brandon S., Dekel D.R., Hassan N.U., Sougrati M.-T. (2021). Understanding how single-atom site density drives the performance and durability of PGM-free Fe–N–C cathodes in anion exchange membrane fuel cells. Mater. Today Adv..

[39] Maldonado S., Stevenson K.J. (2005). Influence of nitrogen doping on oxygen reduction electrocatalysis at carbon nanofiber electrodes. J. Phys. Chem. B.

[40] Lai L., Potts J.R., Zhan D., Wang L., Poh C.K., Tang C., Gong H., Shen Z., Lin J., Ruoff R.S. (2012). Exploration of the active center structure of nitrogen-doped graphene-based catalysts for oxygen reduction reaction. Energy Environ. Sci..

[41] Lin Z., Song M.K., Ding Y., Liu Y., Liu M., Wong C.P. (2012). Three-dimensional nitrogen-doped carbon nanotubes/graphene structure used as a metal-free electrocatalyst for the oxygen reduction reaction. Phys. Chem. Chem. Phys..

[42] Zhao Y., Watanabe K., Hashimoto K. (2012). Self-supporting oxygen reduction electrocatalysts made from a nitrogen-rich network polymer. J. Am. Chem. Soc..

[43] Kramm U.I., Dodelet J.P. (2012). Structure of the catalytic sites in Fe/N/C-catalysts for O_2_-reduction in PEM fuel cells. Phys. Chem. Chem. Phys..

[44] Olson T.S., Pylypenko S., Fulghum J.E., Atanassov P. (2010). Bifunctional Oxygen Reduction Reaction Mechanism on Non-Platinum Catalysts Derived from Pyrolyzed Porphyrins. J. Electrochem. Soc..

[45] Pariiska O., Mazur D., Cherchenko K., Kurys Y., Koshechko V., Pokhodenko V. (2022). Efficient Co-N-C electrocatalysts for oxygen reduction derived from deep eutectic solvents. Electrochim. Acta.

[46] Sgarbi R., Kumar K., Jaouen F., Zitolo A., Ticianelli E.A., Maillard F. (2019). Oxygen reduction reaction mechanism and kinetics on M-NxCy and M@N-C active sites present in model M-N-C catalysts under alkaline and acidic conditions. J. Solid State Electrochem..

[47] Gollasch M., Müller-Hülstede J., Schmies H., Schonvogel D., Wagner P., Dyck A., Wark M. (2021). Elucidating Synergistic Effects of Different Metal Ratios in Bimetallic Fe/Co-N-C Catalysts for Oxygen Reduction Reaction. Catalysts.

[48] Yang L., Cheng D., Xu H., Zeng X., Wan X., Shui J., Xiang Z., Cao D. (2018). Unveiling the high-activity origin of single-atom iron catalysts for oxygen reduction reaction. Proc. Natl. Acad. Sci. USA.

[49] Zhang J., Zhu W., Pei Y., Liu Y., Qin Y., Zhang X., Wang Q., Yin Y., Guiver M.D. (2019). Hierarchically Porous Co–N–C Cathode Catalyst Layers for Anion Exchange Membrane Fuel Cells. ChemSusChem.

[50] Putri Y.M.T.A., Jiwanti P.K., Irkham, Gunlazuardi J., Einaga Y., Ivandini T.A. (2021). Nickel–Cobalt Modified Boron-Doped Diamond as an Electrode for a Urea/H_2_O_2_ Fuel Cell. Bull. Chem. Soc. Jpn..

[51] Hu Z., Xiao Q., Xiao D., Wang Z., Gui F., Lei Y., Ni J., Yang D., Zhang C., Ming P. (2021). Synthesis of Anti-poisoning Spinel Mn–Co–C as Cathode Catalysts for Low-Temperature Anion Exchange Membrane Direct Ammonia Fuel Cells. ACS Appl. Mater. Interfaces.

[52] Kruusenberg I., Ramani D., Ratso S., Joost U., Saar R., Rauwel P., Kannan A.M., Tammeveski K. (2016). Cobalt-Nitrogen Co-doped Carbon Nanotube Cathode Catalyst for Alkaline Membrane Fuel Cells. ChemElectroChem.

